# Probability Weighted Ensemble Transfer Learning for Predicting Interactions between HIV-1 and Human Proteins

**DOI:** 10.1371/journal.pone.0079606

**Published:** 2013-11-18

**Authors:** Suyu Mei

**Affiliations:** Software College, Shenyang Normal University, Shenyang, China; Centro de Biología Molecular Severo Ochoa (CSIC-UAM), Spain

## Abstract

Reconstruction of host-pathogen protein interaction networks is of great significance to reveal the underlying microbic pathogenesis. However, the current experimentally-derived networks are generally small and should be augmented by computational methods for less-biased biological inference. From the point of view of computational modelling, *data scarcity*, *data unavailability* and *negative data sampling* are the three major problems for host-pathogen protein interaction networks reconstruction. In this work, we are motivated to address the three concerns and propose a probability weighted ensemble transfer learning model for HIV-human protein interaction prediction (*PWEN-TLM*), where *support vector machine* (*SVM*) is adopted as the individual classifier of the ensemble model. In the model, *data scarcity* and *data unavailability* are tackled by homolog knowledge transfer. The importance of homolog knowledge is measured by the *ROC-AUC* metric of the individual classifiers, whose outputs are probability weighted to yield the final decision. In addition, we further validate the assumption that only the homolog knowledge is sufficient to train a satisfactory model for host-pathogen protein interaction prediction. Thus the model is more robust against *data unavailability* with less demanding data constraint. As regards with *negative* data construction, experiments show that *exclusiveness of subcellular co-localized proteins* is unbiased and more reliable than *random sampling*. Last, we conduct analysis of overlapped predictions between our model and the existing models, and apply the model to novel host-pathogen PPIs recognition for further biological research.

## Introduction

Accurate mapping of protein interactome is essential to reveal protein functions, biological processes, signal transduction pathways. In recent years, although high throughput experimental techniques have drastically accumulated much knowledge about protein-protein interactions (PPI), the derived PPI networks are far incomplete and noisy [1], [2]. As a good complement to the labour-intensive biological experiments, computational methods can accelerate the reconstruction of PPI networks at low cost [3].

At present most of the existing computational methods are developed for *intra-species* PPI networks reconstruction, e.g. yeast PPI network [3], *Arabidopsis thaliana* PPI network [4], human PPI network [5], etc. As compared to *intra-species* PPI networks reconstruction, *inter-species* host-pathogen PPI networks (the interacting partners are from two different species) reconstruction is faced up with more challenges in that the scale of the host-pathogen PPI networks is generally rather small. Small PPI network results in *data scarcity* that would easily lead to poor generalization ability of computational model. *Data integration* is an effective method to compensate for *data scarcity*. By simultaneously leveraging a catalog of biological feature information, *data integration* can greatly increase the information abundance for sufficient model training. Tastan et al. [6] applied *Random Forest* to integrate the feature information of *binding motif*, *gene expression profile*, *gene ontology*, *sequence similarity*, *post-translational modification*, *tissue distribution* and *PPI network topology* for HIV-human protein interaction prediction. Based on the work, Qi et al. [7] further proposed a semi-supervised multi-task learning method to exploit the weakly labelled data. Dyer et al. [8] combined *protein domain profile*, *sequence k-mer* and *PPI network properties* for HIV-human protein interaction prediction. For another pathogenetic microbe *Plasmodium falciparum*, Dyer et al. [9] combined *protein domain profile*, *gene expression*, *gene ontology* and *gene co-expression* to predict and validate the host-pathogen protein interactions. Wuchty S [10] combined *sequence k-mer*, *interlog*, *gene ontology* and *signal transduction pathways* to predict and validate the protein interactions between *Plasmodium falciparum* and *Homo sapiens*. In the latter two models, the validation information (*gene co-expression*, *signal transduction pathways*, *gene ontology*) was used to manually filter the predicted PPIs. It has been claimed that *gene ontology* (*GO*) is one of the strongest indicators for host-pathogen PPI prediction [6] and *intra-species* PPI prediction [3], [4], [11], [12], [13], [14], [15], [16], [17] among the catalog of feature information. The work [14] explained the reasons why *GO* feature outperformed the other feature information based on the observations: (1) proteins localized in identical *cellular compartments* are more likely to interact than are proteins that reside in spatially distant compartments; (2) proteins that participate in similar *biological processes* or perform similar *molecular functions* are likely to interact. Hence the three aspects of *gene ontology* (*cellular compartments, biological processes* and *molecular functions*) are informative to indicate PPI.

Although *data integration* can simultaneously exploit multiple aspects of biological knowledge, the difficulty in availability of some feature information such as *gene co-expression* poses a great challenge on host-pathogen PPI networks reconstruction [9]. Once the feature information is unavailable for the proteins to be predicted, the *data integration* methods [4], [6], [7], [11], [16] would fail to work. Even for those methods that exploit only one type of non-sequence feature information (e.g. *gene ontology*) [14], *data integration* would also fail to work because the information required for prediction (e.g. *GO* annotations) may be potentially not available. For the reasons, *data integration* model should deliberately take into account the case of *data unavailability* and provide effective solutions to information substitution. Less demanding data constraint helps the model gain wide applicability. Like the other feature information, *structural similarity*, is also a strong indicator of protein-protein interaction. Doolittle et al. [18] exploited the information of protein *structural similarity* to predict host-pathogen PPI. However, the potential unavailability of the *spatial structural information* would likewise restrict the model application. As compared to the costly feature information such as *structural information*, *gene ontology*, *gene co-expression* and *metabolic pathways*, etc., obtaining protein sequence information is less expensive, thus the computational model based on protein sequence only has the least data constraint nearly without the problem of *data unavailability*. Unfortunately, the work [19] argued that protein sequence alone was not sufficient to train a satisfactory model for PPI prediction.

HIV-human PPI prediction can be viewed as a problem of 2-class classification that needs both *positive* data and *negative* data to define the decision function. *Positive* data contains the information of interaction and *negative* data contains the information of non-interaction. Unfortunately, there are far few experimentally derived *negative* data available to computational modelling for host-pathogen PPI networks reconstruction. At present *negative* data construction is a hard-tackling problem and the common method is *random sampling*. *Random sampling* is simple but has the demerits of model uncertainty and potential inclusion of interacting protein pairs. The work [20] proposed one-class *Biclustering* method to mine association rules from the *positive* data for HIV-human PPI prediction. *Biclustering* need not construct the *negative* data, so that the computational modelling is much simplified. However, the model that does not learn the *non-interacting* patterns would run the risk of high rate of *false positive*.

In this work, we are motivated to address the concerns of *data scarcity*, *data unavailability* and *negative data sampling* for HIV-human PPI prediction. To reduce data dependency, we choose *gene ontology* as the only feature information for host-pathogen PPI prediction. Unlike the existing *GO*-based PPI prediction models [3], [4], [6], [7], [11], [12], [13], [14], [15], [16], [17], we attempt to exploit the homolog *GO* information (*GO* annotations from the homologs) to compensate for *data scarcity* and *data unavailability*. We deliberately investigate the assumption that only the homolog *GO* information is sufficient to train a satisfactory model for HIV-human PPI prediction. If the assumption is validated, effective information substitution could make the model more robust against *data unavailability* with less demanding data constraint. To validate the assumption, we conduct three experimental settings, namely the *Optimistic case*, the *Moderate case* and the *Pessimistic case*. The *Optimistic case* assumes that both the target *GO* information (*GO* annotations from the protein itself) and the homolog *GO* information are available for model training and model evaluation. Good performance can indicate that *data scarcity* is properly tackled to a certain degree. The *Moderate case* assumes that the target *GO* information of the test data is not available and the *Pessimistic case* assumes that the target *GO* information of the training data and the test data is not available. If any of the two cases achieves good performance, it can be convincingly concluded that *data unavailability* is well tackled. As regards with *negative data sampling*, we further conduct two experimental settings for each case, one is *random sampling* and the other is *exclusiveness of subcellular co-localized proteins*. All the tasks are implemented by our proposed *p*robability *w*eighted *en*semble *t*ransfer *l*earning *m*odel (*PWEN-TLM*). The target *GO* information and the homolog *GO* information are used to train individual *support vector machine* (*SVM*) and are assigned different weights according to their contributions to the model performance. The merit is that the weights could depress the potential noise from the homolog *GO* information. To investigate the importance of *molecular functions*, *cellular compartments* and *biological processes* (three aspects of *gene ontology*) to HIV-human PPI prediction, the three aspects of the target *GO* information and the homolog *GO* information are used to train three individual classifiers respectively, thus there are totally 6 individual classifiers. The ensemble classifier yields the final decision in the form of probability by linearly weighting the probability outputs of the individual classifiers. For critical model performance estimation, we conduct cross validation, independent test and novel PPI detection on the benchmark HIV-human PPI dataset [21].

## Methods

### Transfer Learning

Transfer learning is a hot research topic in machine learning community. As compared to traditional supervised learning, transfer learning aims at leveraging useful information from auxiliary data. In most cases, the auxiliary data and the target data show different distributions or heterogeneous representations [22]. Especially in bioinformatics field, the biological data from different laboratories are usually subjected to different distributions, heterogeneous representations and noise levels [23]. Thus it is necessary for us to develop sophisticated transfer learning models to exploit useful information from the auxiliary data for the target domain learning. The work [24], [25], [26] proposed several non-parametric multiple kernel learning based transfer learning models (*GO-TLM, MK-TLM* and *MLMK-TLM*) to reduce the risk of *negative knowledge transfer*. In this work, we propose a probability weighted ensemble learning model (*PWEN-TLM*) to transfer the homolog *GO* information to enrich or substitute for the target *GO* information. As compared to multiple kernel learning based transfer learning models, the ensemble based transfer learning method can take full advantages of *SVM* (*support vector machine*) *sparseness* to reduce the computational complexity. The details are described in the section *Probability weighted ensemble learning*.

### *GO* Feature Construction

The homologs are extracted from *SwissProt* 57.3 database [27] using *PSI-BLast* [28]. Here we adopt the *default* parameters setting (e.g. default *E-value* = 10) to enlarge the *GO* term coverage. The *GO* terms are extracted from the latest *GOA* database [29] (114 Release, as of 28 November, 2012). For each protein *i*, we separate the target set of *GO* terms (denoted as 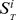) from the homolog set of *GO* terms (denoted as 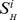), and further divide 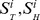 into three subsets corresponding to the three aspects of *gene ontology*, denoted as 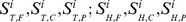, respectively. Here *T* denotes the target protein, *H* denotes the homolog protein, *F* denotes *molecular functions*, *C* denotes *cellular components* and *P* denotes *biological processes*. It is noted that the term *target* here is used to denote the protein itself (comparative to *homolog*), it does not refer to the virus*-targeted* protein. Let capital *I* denote the set of proteins, then the total set of *GO* terms can be defined as follows:
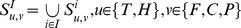
(1)


Based on the denotations, we can formally define the feature vector for each PPI pair (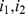) as follows:
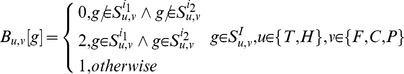
(2)where 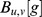 denotes the component *g* of PPI feature vector 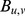 (each PPI pair follows the same feature representation, so we use 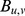 instead of 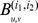 as the general definition). Formula (2) means that if the interacting protein pair shares the same *GO* term *g*, then the corresponding component in the feature vector *B* is set 2; if neither protein in the protein pair (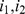) possesses *GO* term *g,* then the value is set 0; otherwise the value is set 1. From the formula, we can see that the above definition is symmetrical, i.e., (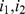) and (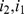) have identical feature representation, thus the order of the proteins in each protein pair does not change the feature representation.

### Probability Weighted Ensemble Learning

*Sparseness* is one of the graceful characteristics of *SVM*, which means that the parameters are optimized on a small *working set* instead of the whole training set [30]. *Kuhn-Tucker Theorem* states that only the training examples that lie on the surface of the optimal hypersphere have their corresponding *Lagrange* parameters non-zero, and the corresponding *Lagrange* parameters are all zero for the remaining examples. The training examples with non-zero *Lagrange* parameters are referred to as *support vectors*. Only the *support vectors* are informative to support the optimal hypersphere and the other data can be discarded. Assuming there are 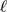 training data, the *working set* that helps define the final decision function generally contains rather small number of data points 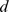, 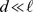, that’s, we only need to compute the kernel matrix on the *working set* (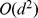) instead of the whole training dataset (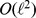), thus the runtime complexity and the space complexity are greatly reduced. In our method, the six independent individual *SVM*s (denoted as 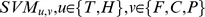) trained by the six feature vectors (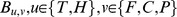) have time complexity 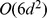, much smaller than the *multiple kernel learning* method 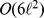.

Traditional two-class labels {−1, +1} are not convenient to reveal the confidence level of the prediction. Probability output is a good alternative to the classical 2-class output and is especially applicable to vote-weighted ensemble learning for the final decisions combination. Platt [31] proposed a method to yield posterior class probability output for binary *SVM* as defined below:
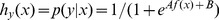
(3)where the coefficient *A* and *B* can be derived from data by cross validation, and *f(x)* is the decision value of binary *SVM*. The final decision function of the ensemble classifier is defined as follows:
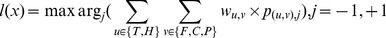
(4)where 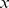 denotes the test protein, 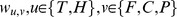 denotes the weight of the individual classifier 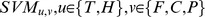 and 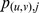 denotes the probability that the individual classifier 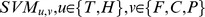 assigns protein 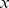 to the jth class. ROC curve [32] is a frequently-used statistical tool to illustrate the predictive performance of 2-class classification. In this work, we use AUC score (area under the ROC curve) to measure the individual SVM weight 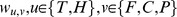:
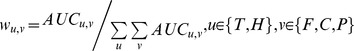
(5)where 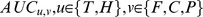 can be derived by 2-fold cross validation on the training set. The individual 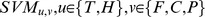 adopts Gaussian kernel defined as follows:
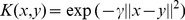
(6)where 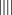
denotes 2-norm of a vector, and the hyperparameter 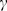 controls the flexibility of the kernel.

### Model Evaluation and Model Selection

We design three experimental settings, namely the *Optimistic case*, the *Moderate case* and the *Pessimistic case*, to validate the assumptions that the homolog *GO* information is useful to tackle the problems of *data scarcity* and *data unavailability*. To formally define the three cases, we first define the following sets:
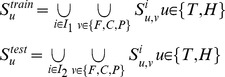
(7)where 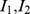 denote the training set and the test set, 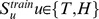 denotes the target *GO* term set and the homolog *GO* term set of the training data, 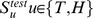 denotes the target *GO* term set and the homolog *GO* term set of the test data. Based on the notations, we can formally define the three cases as follows:
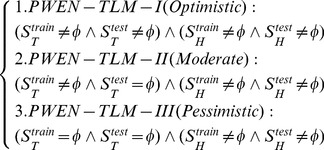
(8)


From the formula, we can see that both the training set and the test set abound in target *GO* information in the *Optimistic* case, the test set contains no target *GO* information in the *Moderate case*, and neither the training set nor the test set contains target *GO* information in the *Pessimistic case.* In the *Moderate case*, we substitute the homolog *GO* information 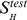 for the missing target *GO* information 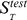. In the *Pessimistic case*, we use the homolog *GO* information (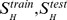) alone.

We conduct model estimation and mode selection by two-level cross validation. The outer 3-fold cross validation is conducted for model estimation and the inner 2-fold cross validation is conducted to derive the weights of individual *SVM* classifiers. For the outer 3-fold cross validation, the dataset is randomly divided into three nearly-even disjoint subsets that have the same distributions as the original dataset (*stratified cross validation*). For each outer fold, one subset is used as test set and the other two subsets are merged as training set, which repeat three times until all data are estimated. Within each outer fold, 2-fold inner cross validation is further conducted for weight derivation on the training set.

HIV-1 protein can be catalogued as Env, Gag, Nef, Pol, Rev, Tat, Vif, Vpr and Vpu [21]. For the sake of critical assessment of model performance, we also conduct several independent tests by treating one catalogue of HIV-1 proteins (e.g. Env) as independent test set and the other catalogues of HIV-1 proteins (e.g. Gag, Nef, Pol, Rev, Tat, Vif, Vpr, Vpu) are merged as training set. In such a way, the independent test is more challenging because the test data (e.g. Env) have no corresponding training data in the training set (e.g. Gag, Nef, Pol, Rev, Tat, Vif, Vpr, Vpu). Wide variance between the test set and the training set helps conduct more critical performance estimation on the proposed model.

The model performance is measured by *Receiver Operating Characteristic* (*ROC*) *AUC* (*Area Under Curve*) (*ROC-AUC*), *Precision recall curve AUC* (*PR-AUC*), *Specificity* (*SP*), *Sensitivity* (*SE*) and *MCC* (*Matthews correlation coefficient*). The performance metrics *SP*, *SE* and *MCC* can be calculated through confusion matrix *M*. By means of the intermediate variables defined as formula (9), we can calculate *SP*, *SE* and *MCC* for each label (*SP_l_*, *SE_l_* and *MCC_l_*) by formula (10), and further calculate the overall accuracy (*Acc*) and the overall MCC (*MCC*) by formula (11).
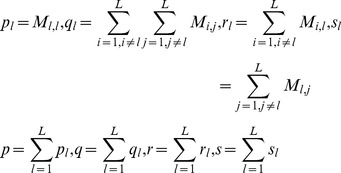
(9)

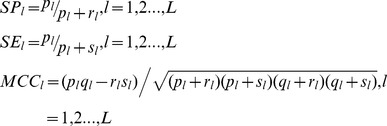
(10)

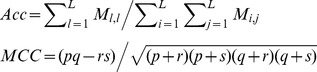
(11)where the confusion matrix 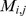 records the counts that class 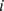 are classified to class 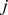 and *L* denotes the number of labels. *AUC* is calculated based on the weighted *SVM* decision values.

## Results

### Data and Materials

The interactions between HIV-1 and human proteins are taken from the database available at http://www.ncbi.nlm.nih.gov/projects/RefSeq/HIVInteractions/ [21]. In order to acquire corresponding *gene ontology* annotations, we map the protein accessions to Uniprot accessions via the id mapping file available at ftp://ftp.uniprot.org/pub/databases/uniprot/current_release/knowledgebase/idmapping/idmapping.dat.gz. After removing duplicate PPIs and putative PPIs, we totally get 3,638 PPIs including 539 Env PPIs, 487 Gag PPIs, 349 Nef PPIs, 272 Pol PPIs, 278 Rev PPIs, 1,101 Tat PPIs, 126 Vif PPIs, 338 Vpr PPIs and 148 Vpu PPIs. All the PPIs are treated as *positive* data. As far, there is no gold-standard *negative* data available for model training and model assessment. How to construct *negative* data is still a challenging problem for PPI prediction. At present, the common practice to generate *negative* data is *random sampling* from the huge protein-protein pair space exclusive of those experimentally derived PPIs. Unbiased as it is, *random sampling* would probably introduce a certain level of noise. For the reason, the work [33] proposed to exclude those subcellular *co-localized* proteins out of the *negative* data (hereinafter called *exclusiveness of subcellular co-localized proteins*), based on the common sense that subcellular *co-localization* is the premise of protein-protein physical interaction. But even so, the method still received criticism that the information about protein subcellular localization is likely to dominate the prediction and thus yields bias. In this work, we will compare the two methods of *negative data sampling* and investigate whether or not *exclusiveness of subcellular co-localized proteins* yields model bias. For simplicity of reference, we call *S1* the dataset with *negative* data exclusive of subcellular *co-localized* proteins, and *S2* the dataset with randomly sampled *negative* data. Dataset *S1* and *S2* both contain 3,638 *positive* data and 3,638 *negative* data.

How to determine the ratio of *positive* data to *negative* data is a second concern to be addressed. The work [7], [8] solved the problem by introducing different ratio of *positive* data to *negative* data (e.g. 1∶1, 1∶100) to train the model. Actually, the true ratio is hard to determine and pooling so large a *negative* data makes little sense to computational modelling. Contrarily, the adverse effect is that extremely unbalanced training data would yield a highly biased model. For the reason, we construct a *negative* data with the same size as the *positive* data. To randomly select a quality and representative *negative* data is a hard and important problem to computational biologists, though maybe not so appealing to experimental biologists. For reliable computational modelling, experimental evidences of *negatome* should be collected and made available to academic use.

Model comparison is a third concern for the reasons: (1) there is no standard benchmark data available for model evaluation and comparison; (2) some *positive* data are outdated and some novel *positive* data are included; (3) *random sampling* of *negative* data yields different training data; (4) there are no identical data partition of cross validation, etc. Hence, what we can do is to conduct critical assessment on the proposed model and conduct a *rough* comparison with other models for biologists’ reference.

### Model Performance Evaluation

#### Cross validation performance evaluation

**Figure 1:**
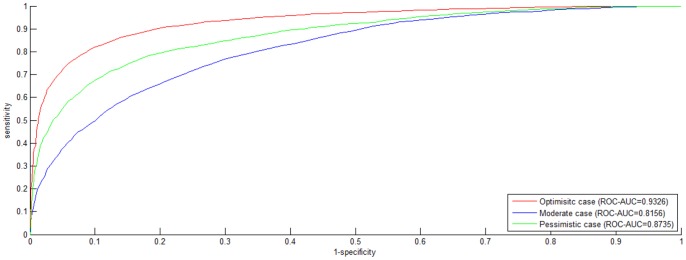
*ROC curve* on *S1* dataset. The negative data is constructed by the negative data sampling method of *exclusiveness of subcellular co-localized proteins*. The *ROC* curves in red, blue and green indicate the performance for the *Optimistic* case, the *Moderate* case and the *Pessimistic* case, respectively.

Dataset *S1* totally contains 7,672 data including 3,638 *positive* data and 3,638 *negative* data. The *ROC curve* for 3-fold cross validation on dataset *S1* is shown in Figure 1, where the *ROC curves* are drawn for the three cases. In the *Optimistic case*, *PWEN-TLM* achieves *ROC*-*AUC* score 0.9326, a little better than *SMLR* (*ROC*-*AUC* score 0.919) [7] that combined 16 catalogs of feature information including *gene ontology*. From Figure 1, we can see that *PWEN-TLM* performs the best in the *Optimistic* case (*AUC* = 0.9326), the second in the *Pessimistic* case (*AUC* = 0.8735) and the worst in the *Moderate* case (*AUC* = 0.8156). The relatively small performance difference between the *Optimistic* case and the *Pessimistic* case (*ROC*-*AUC* score difference = 0.0591) demonstrates that *PWEN-TLM* still works soundly when the target *GO* information is not available, and thus the homolog *GO* information can be treated as an effective substitute for the potentially unavailable target *GO* information. If the protein pair to be predicted contains novel protein, we can choose the model that is trained for the *Pessimistic* case. The results that *PWEN-TLM* performs the worst in the *Moderate* case can be explained that the heterogeneous distribution between 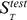 and 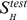 deteriorates the model performance. The performance deterioration reveals that *data unavailability* is an important concern to be addressed for computational modelling. The data integration model *SMLR* [7] did not deliberately dwell on the problem of *data unavailability*.

**Figure 2:**
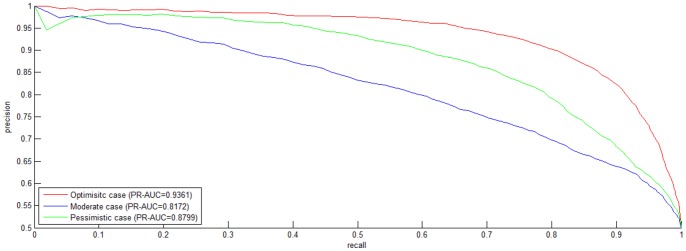
*Precision-Recall (PR) curve* on *S1* dataset. The negative data is constructed by the negative data sampling method of *exclusiveness of subcellular co-localized proteins*. The *PR* curves in red, blue and green indicate the performance for the *Optimistic* case, the *Moderate* case and the *Pessimistic* case, respectively.

Dyer et al. [8] adopted *PR-AUC* (*AUC* of *Precision-Recall Curve*) as the performance metric of HIV-human PPI prediction. In their work, the best *PR-AUC* score among different ratios of *positive* data to *negative* data is 0.707. As compared to *ROC Curve*, *Precision-Recall Curve* is more suited to highly *skewed* (extremely *unbalanced*) data [34]. For comparison, we also plot *Precision-Recall Curve* and indicate the corresponding *PR-AUC* score in Figure 2. As shown in Figure 2, *PWEN-TLM* achieves *PR-AUC* score 0.9361, 0.8172 and 0.8799 in the *Optimistic* case, the *Moderate* case and the *Pessimistic* case, respectively. The *PR-AUC* scores demonstrate that *PWEN-TLM* significantly outperforms the baseline model (*PR-AUC* score 0.707) [7]. By comparing Figure 1 and Figure 2, we can see that there is little difference between *ROC-AUC* score and *PR-AUC* score. The reason is that dataset *S1* is not *skewed* but perfectly *balanced* with 1∶1 ratio of *positive* data to *negative* data. *Skewed* training data is prone to yield a biased model.

*ROC curve* plots the true *positive* rate against the false *positive* rate and *Precision-Recall curve* plots the *precision* against *recall*. Both the curves focus on the reliability of *positive* predictions, but the *negative class* is largely ignored. For 2-class classification, *predictive balance* is an important aspect of model performance. Highly biased predictions are not reliable. From the point of view of biomedical research, true protein-protein non-interaction (i.e. *true negative*) also provides much insight into functional proteomics and drug research. Hence we also report *SP*, *SE*, *MCC* and *Accuracy* for comprehensive survey of model performance. As shown in Table 1, *PWEN-TLM* achieves good *predictive balance* in the *Optimistic* case (*Acc* = 85.62%, *MCC* = 0.7446) and in the *Pessimistic* case (*Acc* = 80.22%, *MCC* = 0.6605). But *PWEN-TLM* shows bias towards the *positive class* in the *Moderate* case (*Acc* = 66.22%, *MCC* = 0.4606, *positive SP* = 0.6015, *negative SE* = 0.3631). Comparatively, *AUC* scores do not detect the bias (*ROC-AUC* score = 0.8156, *PR-AUC* score = 0.8172), implying that the performance metrics of *SP*, *SE*, *MCC* and *Accuracy* are important to model estimation. Summarizing all the performance metrics, we can see that *PWEN-TLM* performs well in the *Optimistic* case and in the *Pessimistic* case. If the target *GO* information of the test data is available, we choose the model trained in the *Optimistic* case; *otherwise,* we choose the model trained in the *Pessimistic* case.

**Table 1:** Cross validation performance estimation on dataset *S1*.

	*PWEN-TLM-I (Optimistic)*			*PWEN-TLM-II (Moderate)*			*PWEN-TLM-III (Pessimistic)*		
	*SP*	*SE*	*MCC*	*SP*	*SE*	*MCC*	*SP*	*SE*	*MCC*
***Positive (interacting)***	0.8774	0.8282	0.7439	0.6015	0.9612	0.5733	0.8160	0.7804	0.6589
***Negative (non-interacting)***	0.8373	0.8843	0.7471	0.9036	0.3631	0.4604	0.7896	0.8241	0.6631
***[AUC;Acc;MCC]***	[0.9326; 85.62%; 0.7446]			[0.8155; 66.22%; 0.4606]			[0.8735;80.22%;0.6605]		

**Figure 3:**
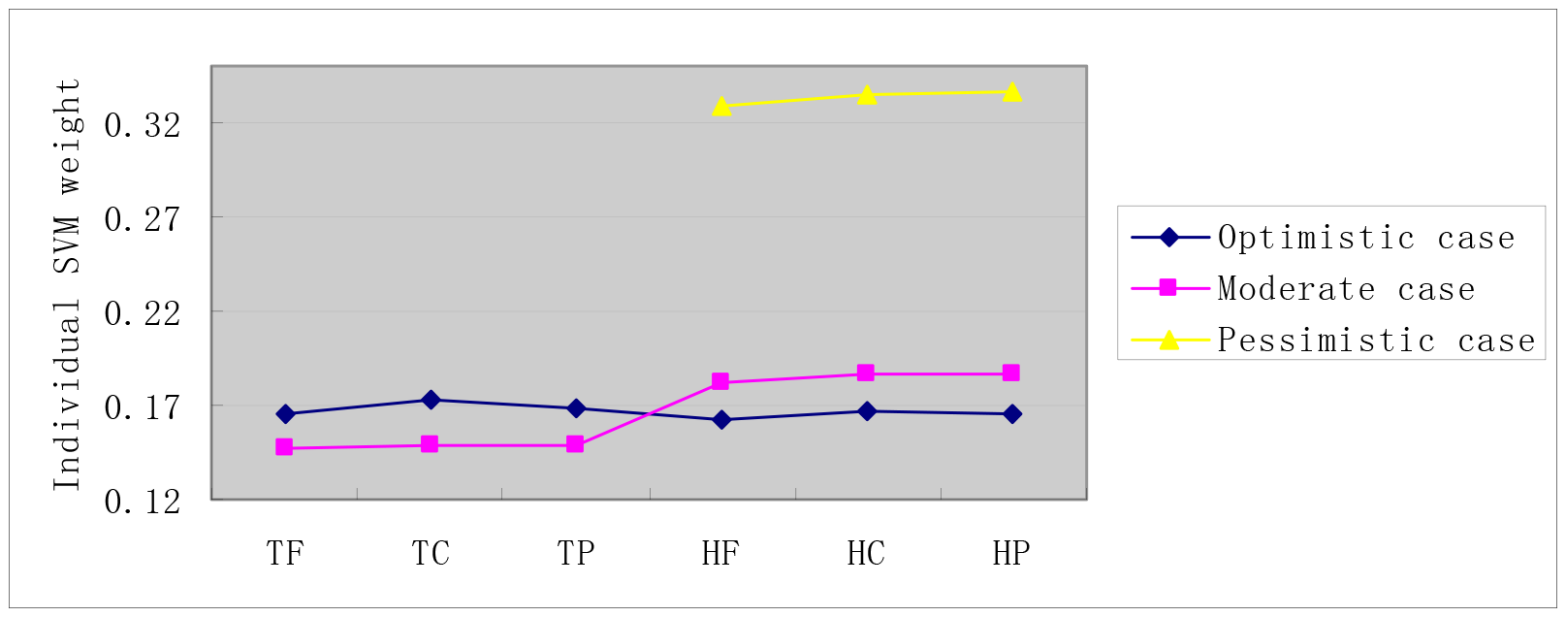
Individual *SVM* weight distribution on *S1* dataset. The negative data is constructed by the negative data sampling method of *exclusiveness of subcellular co-localized proteins*. The horizontal axis is the combination of two sets {T, H} and {F,C, P}. T denotes the target protein, H denotes the homolog protein; F denotes *molecular function*, C denotes *cellular component* and P denotes *biological process*.

To attenuate the noise from the homolog *GO* information, we explicitly investigate the importance of the three aspects of *gene ontology* (*molecular function*, *cellular component*, *biological process*) to HIV-human PPI prediction. As illustrated in Figure 3, the target *GO* information and the homolog *GO* information contribute equivalently to the model performance in the *Optimistic* case. In the *Moderate* case, the target *GO* information unexpectedly makes less contribution than the homolog *GO* information. The result is not surprising, because we substitute the homolog *GO* information 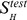 for the missing target *GO* information 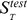 to derive the weights of the target *GO* information. The heterogeneous distribution between 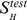 and 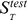 unjustly decreases the importance of the target *GO* information. The three aspects of *gene ontology* unexceptionally make equivalent contributions to the model performance in all the three cases. The *GO* information about *cellular component* does not *predominate* the contributions to model performance, indicating that the *negative* data constructed by *exclusiveness of subcellular co-localized proteins* does not yield predictive bias as worried about.

**Figure 4:**
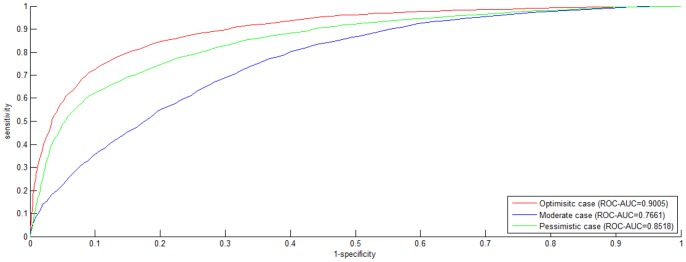
*ROC curve* on *S2* dataset. The negative data is constructed by by the negative data sampling method of *random sampling*. The *ROC* curves in red, blue and green indicate the performance for the *Optimistic* case, the *Moderate* case and the *Pessimistic* case, respectively.

**Figure 5:**
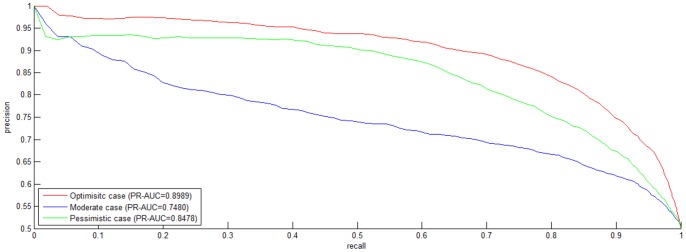
*Precision-Recall (PR) curve* on *S2* dataset. The negative data is constructed by by the negative data sampling method of *random sampling*. The *PR* curves in red, blue and green indicate the performance for the *Optimistic* case, the *Moderate* case and the *Pessimistic* case, respectively.

Dataset *S2* similarly contains 7,672 data including 3,638 *positive* data and 3,638 *negative* data, with the exception to dataset *S1* that the *negative* data are randomly sampled. The *ROC curve* and the *PR curve* are plotted in Figure 4 and Figure 5. Comparing Figure 1 with Figure 4 and Figure 2 with Figure 5, we can see that dataset *S1* achieves higher *ROC-AUC* score and *PR-AUC* score than dataset *S2* for all the three cases. The highest difference of *ROC-AUC* score is 0.0495 and the highest difference of *PR-AUC* score is 0.0692. Table 2 demonstrates the performance metrics of *SP*, *SE*, *MCC* and *Accuracy* on dataset *S2*. Comparing Table 1 and Table 2, we can see that dataset *S1* demonstrates much better predictive balance than dataset *S2*, with highest *MCC* difference 0.1053. The results demonstrate that *exclusiveness of subcellular co-localized protein*s is more reliable to construct a reliable and unbiased classifier than r*andom sampling*.

**Table 2:** Cross validation performance estimation on dataset *S2*.

	*PWEN-TLM-I (Optimistic)*			*PWEN-TLM-II (Moderate)*			*PWEN-TLM-III (Pessimistic)*		
	*SP*	*SE*	*MCC*	*SP*	*SE*	*MCC*	*SP*	*SE*	*MCC*
***Positive (interacting)***	0.8413	0.7988	0.6926	0.5832	0.9560	0.5515	0.7811	0.7622	0.6175
***Negative (non-interacting)***	0.8085	0.8494	0.6966	0.8780	0.3167	0.4143	0.7678	0.7864	0.6204
***[AUC;Acc;MCC]***	[0.9005;82.41%;0.6393]			[0.7661;63.63%;0.4258]			[0.8518;77.43%;0.6188]		

**Figure 6:**
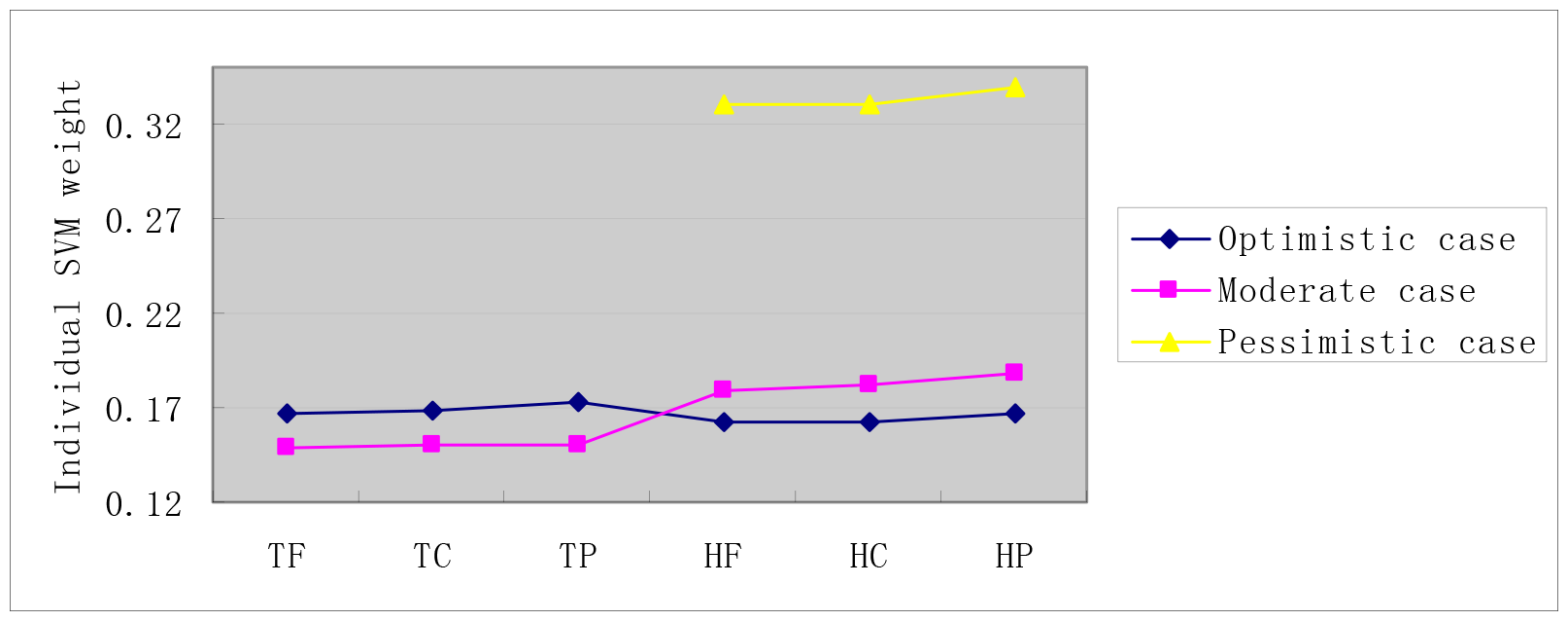
Individual *SVM* weight distribution on *S2* dataset. The negative data is constructed by by the negative data sampling method of *random sampling*. The horizontal axis is the combination of two sets {T, H} and {F,C, P}. T denotes the target protein, H denotes the homolog protein; F denotes *molecular function*, C denotes *cellular component* and P denotes *biological process*.

The weight distribution for the three aspects of *gene ontology* is illustrated in Figure 6. Comparing Figure 3 with Figure 6, we can see that there is little difference of weight distribution between dataset *S1* and dataset *S2*.

#### Independent test performance evaluation

The HIV-human PPI database [18] is catalogued into 9 categories (539 Env PPIs, 487 Gag PPIs, 349 Nef PPIs, 272 Pol PPIs, 278 Rev PPIs, 1,101 Tat PPIs, 126 Vif PPIs, 338 Vpr PPIs and 148 Vpu PPIs). To evaluate the generalization ability, we simply use one catalog of PPI (e.g. Env PPI) as independent test set and other catalogs of PPIs (e.g. Gag, Nef, Pol, Rev, Tat, Vif, Vpr, Vpu) are merged together as *positive* training set. The corresponding *negative* training set is derived for each catalog of HIV protein with the constraints: (1) the *negative* PPIs and the *positive* PPIs are of the same size; (2) the human proteins that are subcellular *co-localized* with the HIV proteins are excluded; (3) the human proteins are randomly sampled. Constraint (2) is based on the above experimental conclusion that *exclusiveness of subcellular co-localized proteins* yields unbiased and better performance. We don’t conduct independent test for the *Moderate* case because of its poor performance in the cross validation performance evaluation.

The experimental results of the independent test are shown in Table 3. We can see that *PWEN-TLM* can recognize most catalogs of HIV-human PPIs with high *recall rate* except one small Pol PPIs (272 PPIs, *Optimistic* 51.84%, *Pessimistic* 54.04%) and one large Tat PPIs (1,101 PPIs, *Optimistic* 52.04%, *Pessimistic* 55.77%). As compared to the generally small overlap between experimental host-pathogen PPIs and predicted host-pathogen PPIs, e.g. 10% overlap between siRNA screen and predictions [7] and 5.29% recall rate (57 PPIs were computationally recognized out of the 1,078 experimental PPIs) [10], the results are considerably promising. From the results, we also see that the *Optimistic* case is unsurprisingly better than the *Pessimistic* case, because the target *GO* information is available. Nevertheless, *PWEN-TLM* still works well in the *Pessimistic* case. The independent test again validates the assumption that the homolog *GO* information alone is sufficient to train a satisfactory HIV-human PPI classifier.

**Table 3:** Independent test performance estimation.

	*env*	*gag*	*nef*	*pol*	*rev*	*tat*	*vif*	*vpr*	*vpu*
	*539*	*487*	*349*	*272*	*278*	*1,101*	*126*	*338*	*148*
***PWEN-TLM-I (Optimistic)***	68.46%	81.34%	88.54%	51.84%	64.75%	52.04%	88.89%	80.77%	66.89%
***PWEN-TLM-III (Pessimistic)***	67.53%	65.91%	77.36%	54.04%	52.88%	55.77%	87.30%	81.66%	66.89%

### Novel PPI Prediction

#### Overlap analysis of predicted interactions between *PWEN-TLM* and the existing models

Overlap analysis of predicted interactions between different computational models is of significance to reveal the confidence and complementariness of predictions. In this work, we investigate the overlap of predictions between *PWEN-TLM* and the latest *bi-clustering* method [20], for the reason that *bi-clustering* has found several supporting evidences from the recent literature. In *bi-clustering* method, there are 180 predicted interactions, among which there are 80 interactions overlapped with the work [6]. As pointed out in the work [20], some predicted interactions have been validated by the recent literatures, e.g. env_gp120:CASP8[83.33%] [35], env_gp120:CD86[83.33%] [36], env_gp120: NOS3[74.67%] [37], env_gp120:SOD2[88.89%] [38,], env_gp120:SRC[78%] [39], env_gp41:MAPK1[77.78%] [40], Gag_Pr55:MAPK1[71.43%] [41], Tat:TNFSF[86.30%]) [42]. The square bracketed percentage following the protein pair denotes the confidence level of predictions.

We apply *PWEN-TLM* to validate the 180 predicted interactions for overlap analysis. Among the HIV-1 proteins, the protein env_gp120 (*Envelope surface glycoprotein gp120, NP_579894.2*) has no reviewed entry in the *UniprotKB* database (http://www.uniprot.org/uniprot/). The target *GO* information of protein env_gp120 can not be retrieved from the database and thus is treated as novel protein in our model. For reliable training, env_gp120 is not included in the training data. Thus the training data is *more stringent* than that of *bi-clustering* method, because it contains no interaction patterns between env_gp120 and human proteins. The 180 interactions predicted by *bi-clustering* method are treated as test data without overlap with the training data.

The experimental results show that *PWEN-TLM* predicts 132 interactions in the *Optimistic case* (File S1) and 165 interactions in the *Pessimistic case* (File S2). Comparing the results of the two cases, we find that *PWEN-TLM* can not recognize most env_gp120 interactions in the *Optimistic case*, but *PWEN-TLM* behaves contrarily very well in the *Pessimistic case*. The results are not surprising because the unreviewed env_gp120 is treated as novel protein (the target *GO* information is treated as *null* and only the homolog *GO* information takes effect). In the *Pessimistic case*, *PWEN-TLM* correctly recognizes all the literature-validated interactions (env_gp120:CASP8[81.63%];env_gp120:CD86[83.51%];env_gp120:NOS3[84.34%];env_gp120:SOD2[70.78%];env_gp120:SRC[79.54%];env_gp41:MAPK1[89.88%];Tat:TNFSF[90.90%]) except Gag_Pr55:MAPK1 [20]. The results once again validate our model assumption that the homolog *GO* information can be effectively exploited to compensat*e for data scarcity* and *data unavailability*. Especially, we can safely draw the conclusion that the homolog *GO* information alone is sufficient to train a satisfactory model for HIV-human PPI prediction. We can see that *PWEN-TLM* has less demanding data constraint and hardly fails to work even in the worst case (the *Pessimistic* case). As long as *GO* annotated homologs can be retrieved, *PWEN-TLM* can convincingly predict the protein pairs that contain novel proteins. It is noted that although the model is trained without env_gp120 interaction patterns, the env_gp120*-*related interactions are still soundly recognized, which implies that *PWEN-TLM* has good generalization ability.

Besides the validation of the 180 predicted interactions, we also validate against *PWEN-TLM* the 80 overlapped interactions between the two work [6], [20]. The results show that *PWEN-TLM* predicts 46 interactions in the *Optimistic case* (**File S3**) and 61 interactions in the *Pessimistic case* (**File S4**). From the results, we can see that *PWEN-TLM* narrows down the predictions and thus is relatively more conservative than the *bi-clustering* method [20]. Conservative prediction has the merit of low *false positive* rate but meanwhile has the demerit of missing some true interactions (e.g. Gag_Pr55:MAPK1). From the 8 literature-validated interactions, only one unrecognized interaction is acceptable.

#### Predicted interactions with peripheral human proteins

**Figure 7:**
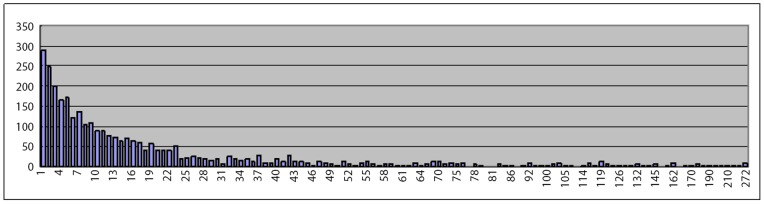
Degree distribution of the HIV-targeted human proteins in human PPI network. The horizontal axis denotes protein degree and the vertical axis denotes the number of proteins that possess that degree.

In addition to validating the interactions predicted by the existing models, we also independently apply *PWEN-TLM* to detect novel HIV-human PPIs for further biological research. To narrow down the scope of potential HIV-targeted human proteins, we first statistically investigate the way that HIV proteins attack the human PPI network. Some diseases, like lung squamous cell carcinoma [43], are prone to attack the densely-connected human proteins (hub proteins). Here we attempt to acquire the knowledge about the behaviour that HIV-1 attacks the human PPI network. We can calculate the degree distribution of the HIV-targeted human proteins from HPRD database (http://hprd.org/) [44]. The degree distribution of the HIV-targeted human proteins in human PPI network is plotted in Figure 7, where the *horizontal axis* denotes the protein degree and the *vertical axis* denotes the number of proteins possessing that degree. From Figure 7, we can intuitively see that the number of HIV-targeted human proteins exponentially decreases with protein degree. It can be inferred from the figure that the HIV proteins are prone to target the peripheral human proteins. For the sake, we choose the peripheral human proteins as test candidates. For each type of HIV proteins, we randomly choose 400 *distinct* human proteins with *lowest degree* (*e.g. degree = 1, 2, 3*) that do not occur in dataset *S1*. The predicted results are shown in File S5 (*Optimistic case*) and File S6 (*Pessimistic case*). Since literature could offer very sparse direct information about the interactions we are concerned about, we analyse the predicted interactions based on the study of *gene ontology*.

#### Interactions with env_gp160

Among the 400 human proteins, *PWEN-TLM* predicts 64 interactions with env_gp160 (P04578) in the *Optimistic case* (**File S5**) and 66 interactions in the *Pessimistic case* (**File S6**). After filtering the weak interactions (probability within [0.5, 0.6]), there are 44 interactions in the *Pessimistic case* and 45 interactions in the *Optimistic case*. Take the *Optimistic case* for example, Table 4 clusters the interacting human partners according to *GO* terms (see Table 4 *Main cluster of interacting human partners*). From Table 4, we can see that env_gp160 mainly interacts with the host *membrane* proteins (*GO:0016020, GO:0016021, GO:0005886*), and the interacting human partners are mainly involved in the *biological processes of metabolic process* (*GO:0044267*), *post-translational modification* (*GO:0043687, GO:0006486*), *transport* (*GO:0006810*), *host immune response* (*GO:0006954, GO:0045087*), etc. From the aspect of *molecular functions*, env_gp160 mainly affects host protein *binding activity* (*GO:0005515*), *transferase activity* (*GO:0016740*), etc. From the analysis of *gene ontology*, we can see that the interactions with env_gp120 may affect the *metabolic process*, *molecule transfer*, *binding activity* of the host proteins and may also activate the *host immune response*.

**Table 4:** Predicted interactions between env_gp160 and human proteins.

GOcategory	Predicted interacting human partners			
	GO term	GO description	Rate	Main cluster of interacting human partners
**Biological** **process**	GO:0044267	cellular proteinmetabolic process	23%	Q9NR34[0.91];Q5I7T1[0.67];Q9NYU2[0.92];Q8N3T1[0.71];Q9NY97[0.86];O43173[0.70];Q13454[0.72];Q9BV94[0.81];O60476[0.91];Q8IUC8[0.70]
	GO:0043687	post-translationalproteinmodification	23%	Q9NR34[0.91];Q5I7T1[0.67];Q9NYU2[0.92];Q8N3T1[0.71];Q9NY97[0.86];O43173[0.70];Q13454[0.72];Q9BV94[0.81];O60476[0.91];Q8IUC8[0.70]
	GO:0006486	proteinglycosylation	18%	Q9NR34[0.91];Q5I7T1[0.67];Q9NYU2[0.92];Q8N3T1[0.71];Q9NY97[0.86];O43173[0.70];O60476[0.91];Q8IUC8[0.70]
	GO:0006457	protein folding	16%	Q9UDY4[0.79];Q9NYU2[0.92];O14967[0.87];O60884[0.79];P30414[0.78];Q14696[0.81];Q9BV94[0.81]
	GO:0006810	transport	9%	P13866[0.75];Q01650[0.86];Q13454[0.72];>O75947[0.73]
	GO:0006954	inflammatoryresponse	9%	O43916[0.87];Q96E93[0.78];Q9NYK1[0.62];Q9H293[0.62]
	GO:0045087	innate immuneresponse	7%	Q96E93[0.78];Q9NYK1[0.62];Q9NY25[0.61]
**Cellular** **component**	GO:0016020	membrane	77%	O43916[0.87];Q86Z14[0.72];Q9NR34[0.91];Q9UDY4[0.79];O75509[0.63];P13866[0.75];Q96E93[0.78];Q8IXI1[0.82];Q5I7T1[0.67];O14967[0.87];Q8N3T1[0.71];Q01650[0.86];P41732[0.63];O75096[0.61];O60884[0.79];Q9NY97[0.86];P30414[0.78];O43173[0.70];P06126[0.82];Q9NYK1[0.62];P04062[0.79];Q13454[0.72];P43626[0.66];Q9P035[0.89];Q9NY25[0.61];O75947[0.73];Q9H293[0.62];Q6UW60[0.69];P23435[0.65];Q9BV94[0.81];O14548[0.71];O60476[0.91];[0.70];P09669[0.77]
	GO:0016021	integral tomembrane	57%	O43916[0.87];Q86Z14[0.72];Q9NR34[0.91];O75509[0.63];P13866[0.75];Q96E93[0.78];Q8IXI1[0.82];Q5I7T1[0.67];O14967[0.87];Q8N3T1[0.71];Q01650[0.86];P41732[0.63];O75096[0.61];Q9NY97[0.86];O43173[0.70];P06126[0.82];Q9NYK1[0.62];Q13454[0.72];P43626[0.66];Q9P035[0.89];Q9NY25[0.61];Q6UW60[0.69];O60476[0.91];Q8IUC8[0.70];P09669[0.77]
	GO:0005886	plasma membrane	32%	Q86Z14[0.72];Q9UDY4[0.79];O75509[0.63];P13866[0.75];Q96E93[0.78];Q8IXI1[0.82];Q5I7T1[0.67];Q01650[0.86];O75096[0.61];P06126[0.82];Q9NYK1[0.62];P43626[0.66];Q9NY25[0.61];Q14696[0.81]
	GO:0000139	Golgi membrane	18%	O43916[0.87];Q9NR34[0.91];Q8N3T1[0.71];Q9NY97[0.86];O43173[0.70];Q9NYK1[0.62];O60476[0.91];Q8IUC8[0.70]
**Molecular** **function**	GO:0016787	hydrolase activity	20%	Q9NR34[0.91];Q8IXI1[0.82];O00754[0.85];P04062[0.79];P04746[0.63];Q9NTJ4[0.74];Q9NRW3[0.67];Q6UW60[0.69];O60476[0.91]
	GO:0016740	transferase activity	18%	O43916[0.87];Q08188[0.81];Q5I7T1[0.67];Q9NYU2[0.92];Q8N3T1[0.71];Q9NY97[0.86];O43173[0.70];Q8IUC8[0.70]
	GO:0005515	protein binding	18%	Q9UDY4[0.79];O75509[0.63];P13866[0.75];Q8IXI1[0.82];O75096[0.61];P13667[0.87];P06126[0.82];P43626[0.66]
	GO:0051082	unfolded proteinbinding	11%	Q9UDY4[0.79];Q9NYU2[0.92];O14967[0.87];O14657[0.61];O60884[0.79]

Illustrations:

[1] Rate denotes that the cluster of interacting human proteins possessing the same corresponding GO term accounts for the total predicted env_gp160-interacting human proteins;

[2] Q9NR34[0.91] denotes that protein env_gp160 is predicted to interact with the human protein P01024 with 0.81 confidence;

#### Interactions with Rev

Among the 400 human proteins, *PWEN-TLM* predicts 37 interactions with Rev (P04618) in the *Optimistic case* and 54 interactions in the *Pessimistic case* (probability>0.6). From Table 5, we can see that Rev mainly interacts with the host *nucleus* proteins (*GO:0005634*) and *cytoplasm* proteins (*GO:0005737*), and participates in the *biological processes* of *viral reproduction* (*GO:0010467, GO:0019083, GO:0016032, GO:0006355*), *viral mRNA translation* (*GO:0016071, GO:0006413, GO:0006414*), etc. These predicted interactions indicate that Rev plays important roles in *viral mRNA transcription* and *mRNA translation into viral proteins*.

**Table 5:** Predicted interactions between Rev and human proteins.

GOcategory	Predicted interacting human partners			
	GO term	GO description	Rate	Main cluster of interacting human partners
**Biological** **process**	GO:0010467	gene expression	14%	Q9P2I0[0.87];P46781[0.90];P62899[0.90];Q9Y3U8[0.86];P62280[0.82]
	GO:0019083	viral transcription	11%	P46781[0.90];P62899[0.90];Q9Y3U8[0.86];P62280[0.82]
	GO:0016071	mRNA metabolicprocess	11%	P46781[0.90];P62899[0.90];Q9Y3U8[0.86];P62280[0.82]
	GO:0016032	viral reproduction	11%	P46781[0.90];P62899[0.90];Q9Y3U8[0.86];P62280[0.82]
	GO:0006355	regulation oftranscription,DNA-dependent	11%	Q9NRC8[0.61];O00472[0.60];Q92925[0.64];Q13342[0.65]
	GO:0006413	translationalinitiation	11%	P46781[0.90];P62899[0.90];Q9Y3U8[0.86];P62280[0.82]
	GO:0006414	translationalelongation	11%	P46781[0.90];P62899[0.90];Q9Y3U8[0.86];P62280[0.82]
**Cellular** **component**	O:0005634	nucleus	41%	Q9H1A4[0.69];Q9P2I0[0.87];Q99877[0.82];Q8IX01[0.62];Q8WWL7[0.70];Q9NRC8[0.61];O00472[0.60];Q9NYP9[0.66];Q92925[0.64];Q9H668[0.66];Q13342[0.65];Q15003[0.67];Q8NDV3[0.74];Q13601[0.76];O15523[0.63]
	GO:0005737	cytoplasm	38%	Q96C10[0.74];P09972[0.63];Q9UBB4[0.76];Q08188[0.68];P46781[0.90];Q06210[0.70];Q01650[0.75];Q9NRC8[0.61];Q9NYP9[0.66];Q9Y3U8[0.86];Q13342[0.65];Q15003[0.67];Q13601[0.76];O15523[0.63]
	GO:0005829	cytosol	30%	Q9H1A4[0.69];P09972[0.63];Q9UBB4[0.76];Q8IXI1[0.72];P46781[0.90];Q06210[0.70];Q01650[0.75];P62899[0.90];Q9Y3U8[0.86];Q9BU89[0.66];P62280[0.82]
	GO:0005730	nucleolus	22%	P46781[0.90];Q8WWL7[0.70];Q9NRC8[0.61];O00472[0.60];Q9H668[0.66];Q9Y3U8[0.86];Q13342[0.65];Q13601[0.76]
**Molecular** **function**	GO:0005515	protein binding	35%	Q96C10[0.74];P09972[0.63];Q9UBB4[0.76];P13866[0.64];Q9P2I0[0.87];Q8IXI1[0.72];P46781[0.90];Q8WWL7[0.70];Q9NRC8[0.61];P06126[0.70];Q9H668[0.66];Q15003[0.67];Q9BU89[0.66]
	GO:0003723	RNA binding	24%	Q96C10[0.74];Q9P2I0[0.87];Q9Y6V7[0.63];P46781[0.90];Q8IX01[0.62];P62899[0.90];Q13601[0.76];P62280[0.82];O15523[0.63]
	GO:0003677	DNA binding	16%	96C10[0.74];Q99877[0.82];Q9H668[0.66];Q13342[0.65];Q8NDV3[0.74];O15523[0.63]

#### Interactions with Vpr

Similarly, the predicted interactions with Vpr (Q77YF9) are shown in Table 6 (18 predicted interactions with probability greater than 0.6). From the results we can see that Vpr mainly affects the *host cell cycle* (*GO:0007049, GO:0051301, GO:0007067, GO:0030261, GO:0007126*) and the *regulation of DNA transcription* (*GO:0006355*). The predicted interactions are consistent with our prior knowledge about HIV-1 Vpr proteins.

**Table 6:** Predicted interactions between Vpr and human proteins.

GOcategory	Predicted interacting human partners			
	GO term	GO description	Rate	Main cluster of interacting human partners
**Biological** **process**	GO:0007049	cell cycle	22%	Q9H1A4[0.63];Q8WWL7[0.72];Q15003[0.61];Q8NDV3[0.82]
	GO:0051301	cell division	17%	Q9H1A4[0.63];Q8WWL7[0.72];Q15003[0.61]
	GO:0007067	mitosis	11%	Q9H1A4[0.63];Q15003[0.61]
	GO:0022904	respiratory electrontransport chain	17%	O75947[0.64];O14548[0.74];P09669[0.77]
	GO:0030261	chromosomecondensation	11%	Q15003[0.61];Q8NDV3[0.82]
	GO:0006355	regulation oftranscription,DNA-dependent	11%	P58012[0.61];Q13342[0.67]
	GO:0007126	meiosis	11%	Q8WWL7[0.72];Q8NDV3[0.82]
**Cellular** **component**	GO:0005634	nucleus	50%	Q9H1A4[0.63];Q9UDY4[0.62];Q99877[0.64];Q8WWL7[0.72];P58012[0.61];Q9H668[0.61];Q13342[0.67];Q15003[0.61];Q8NDV3[0.82]
	GO:0016020	membrane	44%	Q9UDY4[0.62];Q96E93[0.61];Q8IXI1[0.64];P30414[0.69];P04062[0.64];O75947[0.64];O14548[0.74];P09669[0.77]
	GO:0005730	nucleolus	22%	Q9UDY4[0.62];Q8WWL7[0.72];Q9H668[0.61];Q13342[0.67]
	GO:0005737	cytoplasm	22%	Q9UDY4[0.62];Q13342[0.67];Q15003[0.61];Q5SW79[0.68]
**Molecular** **function**	GO:0005515	protein binding	39%	Q9UDY4[0.62];Q8IXI1[0.64];Q8WWL7[0.72];P58012[0.61];Q9H668[0.61];Q15003[0.61];Q5SW79[0.68]
	GO:0003677	DNA binding	28%	Q99877[0.64];P58012[0.61];Q9H668[0.61];Q13342[0.67];Q8NDV3[0.82]
	GO:0003700	sequence-specific DNAbinding transcriptionfactor activity	11%	P58012[0.61];Q13342[0.67]

#### Interactions with other HIV-1 proteins

The predicted interactions with other HIV-1 proteins (Gag, Pol, Tat, Vpu, Nef, Vif) are shown in **File S5** and **File S6**. The experimental results show that Gag mainly interacts with the human proteins that participate in the *biological processes* of *signal transduction* (*GO:0007165*, the interacting partners include *O75509[0.67]; Q9NYK1[0.69]; Q9NY25[0.63]; O60609[0.61]*), *innate immune response* (*GO:0045087, Q96E93[0.64]; Q9NYK1[0.69]; Q9NY25[0.63]*), *apoptotic process* (*GO:0006915, P09972[0.67];O75509[0.67];Q8IXI1[0.81]*), etc. Tat mainly affects the *regulation of transcription* (*GO:0006355, Q9NRC8[0.67]; O00472[0.74]; Q92925[0.72]; Q13342[0.80]*), *host cell defense response to virus* (*GO:0051607, Q96C10[0.67]; Q9NYK1[0.70]; Q9NRW3[0.69]*), etc. Vpu mainly interacts with the human proteins of *transport activity* (*GO:0006810, P13866[0.62]; O75947[0.86]*), *receptor activity* (*GO:0004872, Q96E93[0.70]; P22897[0.61]), cell death* (*GO:0008219, Q9UBB4[0.82]; P04062[0.78]*), etc. Full interactions are shown in **File S5** and **File S6**.

## Discussion

*Data scarcity*, *data unavailability* and *negative data sampling* are the three major concerns to be addressed for the computational reconstruction of HIV-human PPI networks. At present feature-level data integration is still the major effective method to compensate for *data scarcity*, but potential unavailability of some feature information is likely to make the existing data integration methods fail to work. In this work, we are motivated to develop a less data-demanding computational model for HIV-human PPI prediction that hardly fails to work in most cases. We investigate the assumption that the homolog *GO* information is useful to well tackle the problems of *data scarcity* and *data unavailability*. To fulfil the motivation and assumption, we propose a probability weighted ensemble transfer learning model for HIV-human PPI prediction (*PWEN-TLM*). In this model, *gene ontology* is the only feature information used for model training and model evaluation. The target *GO* information and the homolog *GO* information are separately extracted to cope with *data unavailability*, and the three aspects of *gene ontology* are further separated to evaluate their contributions to the model performance. The contributions are measured in terms of weights by *ROC-AUC* performance metric of the individual classifiers. The weights of the homolog *GO* information play the role of enhancing *positive knowledge transfer* and depressing *negative knowledge transfer*.

To validate the assumption that the homolog *GO* information is effective to enrich or substitute for the target *GO* information, we conduct three experimental settings, namely the *Optimistic* case, the *Moderate* case and the *Pessimistic* case. The latter two cases take into account the unavailability of the target *GO* information. 3-fold cross validation and independent test are used to evaluate the model performance. The performance measured by multiple metrics (*ROC-AUC*, *PR-AUC*, *MCC*, *SP*, *SE* and *Accuracy*) show that *PWEN-TLM* performs well in the *Optimistic* case and in the *Pessimistic* case. The sound performance in the *Optimistic* case demonstrates that the homolog *GO* information is useful to solve the problem of *data scarcity* by enriching the target *GO* information. The good performance in the *Pessimistic* case shows that the homolog *GO* information is an effective substitute for the target *GO* information to solve the problem of *data unavailability*.

*Negative data sampling* is another important concern to be addressed for HIV-human PPI prediction. In this work, we have compared *exclusiveness of subcellular co-localization* to *random sampling*. We find that the *GO* information about *cellular components* makes equivalent contributions to the model performance as the *GO* information about *biological processes* and *molecular functions* does. This result shows that *exclusiveness of subcellular co-localized proteins* outperforms *random sampling* without introducing model bias.

Lastly, we apply *PWEN-TLM* to novel HIV-human PPIs detection. The overlap analysis of the predictions between *PWEN-TLM* and the existing models show that *PWEN-TLM* can recognize most of the literature-validated interactions and is relatively more conservative than the *bi-clustering* method. We also report some novel interactions for further biological research. The analysis based on g*ene ontology* shows that the information revealed by the predicted interactions is consistent with our prior knowledge about the HIV-1 proteins.

## Supporting Information

File S1 **Text file contains the overlapped predictions between** ***PWEN-TLM*** **and** ***Bi-clustering*** **** [**20**] **(** ***Optimistic case*** **).**
(TXT)

File S2 **Text file contains the overlapped predictions between** ***PWEN-TLM*** **and** ***Bi-clustering*** **** [**20**] **(** ***Pessimistic case*** **).**
(TXT)

File S3 **Text file contains the overlapped predictions among** ***PWEN-TLM*** **,** ***Bi-clustering*** **** [**20**] **and the method** [**6**] **(** ***Optimistic case*** **).**
(TXT)

File S4 **Text file contains the overlapped predictions among** ***PWEN-TLM*** **,** ***Bi-clustering*** **** [**20**] **and the method** [**6**] **(** ***Pessimistic case*** **).**
(TXT)

File S5 **Text file contains the predictions between HIV-1 and peripheral human proteins (** ***Optimistic case*** **).**
(TXT)

File S6 **Text file contains the predictions between HIV-1 and peripheral human proteins (** ***Pessimistic case*** **).**
(TXT)
